# Geographic Variability in the Association between Socioeconomic Status and BMI in the USA and Canada

**DOI:** 10.1371/journal.pone.0099158

**Published:** 2014-06-16

**Authors:** Alexandre Lebel, Yan Kestens, Christelle Clary, Sherri Bisset, S. V. Subramanian

**Affiliations:** 1 Evaluation Platform on Obesity Prevention, Quebec Heart and Lung Institute, Laval University, Quebec City, Quebec, Canada; 2 Department of Social and Behavioral Sciences, Harvard School of Public Health, Boston, Massachusetts, United States of America; 3 Graduate School of Land Management and Regional Planning, Laval University, Quebec City, Quebec, Canada; 4 CHUM Research Center, Montreal, Canada; 5 Department of Social and Preventive Medicine, University of Montreal, Montreal, Quebec, Canada; 6 Faculty of Nursing Science, Laval University, Quebec City, Quebec, Canada; University of Pennsylvania School of Medicine, United States of America

## Abstract

**Objective:**

Reported associations between socioeconomic status (SES) and obesity are inconsistent depending on gender and geographic location. Globally, these inconsistent observations may hide a variation in the contextual effect on individuals' risk of obesity for subgroups of the population. This study explored the regional variability in the association between SES and BMI in the USA and in Canada, and describes the geographical variance patterns by SES category.

**Methods:**

The 2009–2010 samples of the Behavioral Risk Factor Surveillance System (BRFSS) and the Canadian Community Health Survey (CCHS) were used for this comparison study. Three-level random intercept and differential variance multilevel models were built separately for women and men to assess region-specific BMI by SES category and their variance bounds.

**Results:**

Associations between individual SES and BMI differed importantly by gender and countries. At the regional-level, the mean BMI variation was significantly different between SES categories in the USA, but not in Canada. In the USA, whereas the county-specific mean BMI of higher SES individuals remained close to the mean, its variation grown as SES decreased. At the county level, variation of mean BMI around the regional mean was 5 kg/m^2^ in the high SES group, and reached 8.8 kg/m^2^ in the low SES group.

**Conclusions:**

This study underlines how BMI varies by country, region, gender and SES. Lower socioeconomic groups within some regions show a much higher variation in BMI than in other regions. Above the BMI regional mean, important variation patterns of BMI by SES and place of residence were found in the USA. No such pattern was found in Canada. This study suggests that a change in the mean does not necessarily reflect the change in the variance. Analyzing the variance by SES may be a good way to detect subtle influences of social forces underlying social inequalities.

## Background

The prevalence of adult obesity has risen significantly in many countries over the last decades with increases in average body mass index (BMI, kg/m^2^) being greatest in wealthier countries [1], [2]. The mean worldwide BMI has increased at a rate of approximately 0.5 kg/m^2^ per decade, reaching 1 kg/m^2^ per decade in wealthier nations like the USA and Canada [2]. USA and Canada have among the highest average BMI [2] and the USA has a higher prevalence between the two [3]–[5]. However, trends in obesity prevalence are similar between the two countries, increasing from 15% in the 1970's to 32% in 2005 in the USA, and from 12% to 29% in Canada [6]. Differences in the SES-BMI relationships have also been reported by geographic location [3], [7], with respect to a shift of the burden of obesity towards lower SES groups in developing countries with increasing gross national products [3], [8]. Although this shift in burden is not always clear in the USA and Canada, the difference in prevalence is obvious where Canadians tend to be less obese for both genders and all age categories [5].

Reviews addressing the association between socioeconomic status (SES) and obesity report inconsistent findings [7], [9], [10]. The most consistent finding is the inverse social gradient for obesity in women in the developed world [11]. The USA and Canada are no exception; obesity prevalence is not consistently associated to educational attainment, although prevalence tends to be lower among college graduates in both countries. Income has been found to be negatively associated with obesity, for women [12], [13], but non-significant or even positive for men [14]. Racial disparities have been observed in the USA only, with higher prevalence among the Black population [13], [15].

These inconsistent findings of associations of obesity with gender, race, and SES reflect the complexity of the phenomena, which is multifaceted, multilevel, and evolving [16]. The obesity epidemic may not only be a biological issue, but may be linked to the social status of individuals and their interaction with the environment, which is also unequally distributed across space [17], [18]. Prior studies largely assumed the variance in the SES-obesity associations to be constant, such that the amount of variation in BMI was meant to be the same within all SES groups and uniformly distributed in space. The possibility that these assumptions may be erroneous and thus requiring alternative methodologies, has been raised [11], [19], [20] whereby improved modeling strategies are frequently identified as one possible solution [21], [22]. Analyzing BMI variances specifically for each SES category within a set of hierarchical geographic groupings may reveal subgroups of the population for which the distribution of BMI is distinct (*e.g.* gender-SES categories). Additionally, between and within countries comparisons provide an opportunity to study the moderating effect of social context on the relationship between SES and obesity with respect to public health interventions and/or social policies [23]. Although some research has reported geographic differences in mean BMI between regional contexts for both countries [13], [24], few studies have attempted to explore these aspects [6], [25], and none have modeled the heterogeneity of the SES-BMI associations at the individual-level. Analyzing the variability of the mean BMI by socioeconomic status at multiple geographical levels can help to disentangle the individual effect (who we are) from the contextual effect (where we are); a time-honored conundrum that is widely recognized, but not well understood [26]–[29].

In order to shed light on these issues, this study analyzed and compared SES-BMI associations in the USA and in Canada for both women and men. Specific objectives were to: 1) describe how variance in BMI is distributed at the individual, regional, and subnational levels, 2) globally assess and compare the SES-BMI associations between the USA and Canada, 3) describe geographic variations in BMI within countries, and 4) characterize the geographic patterns of BMI variance by SES group.

## Methods

### Data sources

The 2009 and 2010 samples from the Behavioral Risk Factor Surveillance System (BRFSS, publicly available) and the Canadian Community Health Survey (CCHS, master files only available in Statistics Canada facilities) were used. Both surveys 1) provide a large cross-sectional and nationally representative sample of the non-institutionalized civilian population, 2) collect self-reported information, and 3) produce age-gender adjusted weights based on similar sample strategies. Detailed documentation is available for the BRFSS [30] and the CCHS [31]. Variables are comparable since measurements are either identical (e.g. BMI) or very similar (e.g. household income).

### Study populations and samples size

The study population includes adults from both the USA and Canada. Table 1 presents the sample size for the BRFSS and CCHS. Observations with missing information regarding BMI, age, education, race, household size were removed as well as all pregnant women (10.4% in the USA and 13.8% in Canada). Observations for which income information was missing were kept by using a missing income category. Mean BMI for excluded individuals was the same for all categories (average difference less than 0.1 kg/m^2^) except for Canadian women for whom the mean BMI was 0.85 kg/m^2^ higher. Study samples were reduced to participants living in contiguous and continental states in the USA, and in the 10 provinces of Canada – thereby excluding territories.

**Table 1:** Individuals and spatial units frequency for the BRFSS and CCHS survey, 2009 and 2010.

USA - BRFSS 2009&2010			CANADA - CCHS 2009&2010		
Units	Frequency	Percent	Units	Frequency	Percent
Sample size	883,682	100%	Sample size	113,796	100%
Removed	92,291	10.4%	Removed	15,746	13.8%
Men	309,732	39.1%	Men	44,665	39.3%
Women	481,659	60.9%	Women	53,385	46.9%
States	49	**-**	Provinces	10	**-**
Counties	2284	**-**	Health regions	114	**-**

**BRFSS** = *Behavioral Risk Factor Surveillance System.*

**CCHS** = *Canadian Community Health Survey.*

### Hierarchical structure

Observations were structured according to a three-level hierarchy, *i.e.* individuals, counties, and states in the USA, and individuals, health regions, and provinces in Canada. To facilitate reading when comparing countries, we re-labeled the hierarchy as individuals nested in regions nested in subnational units. Although regions and subnational units generally fulfill similar administrative functions in both countries, comparing results based on these units between countries may be questioned. More precisely, while the size of countries are about the same (about 10 million km^2^), population density in the USA is well over 30 people/km^2^, but barely reaches 4 people/km^2^ in Canada. In these contexts, how distance and the notion of region are considered or operationalized may produce a very different geographical structure. As a result, the sample size and the number of regions within subnational units are systematically higher in the USA, while the area of the units is systematically larger in Canada. For these reasons, we did not merge the BRFSS and CCHS databases and kept independent statistical models for both countries. This procedure provides a robust analytical precision whereby interpretation of results are within a country. We applied exactly the same analytical procedure for each database and produced generalizable and comparable regional BMI estimates. However, comparing second and third level variance metrics between countries might not be as straightforward, but still allowed global comparisons of geographic patterns between countries.

### Outcome

The primary outcome was body mass index (BMI), calculated as weight in kilograms divided by height squared in meters (BMI = kg/m^2^). Individuals with a BMI lower than 12 or higher than 70 were considered as extreme outliers or reporting errors and were discarded from the final sample (<1%). Table 2 presents summary statistics of the BMI distribution for the four sub-samples of interest, that is, women and men in the USA and in Canada. Overall, mean BMI was higher for Americans than for Canadians, and lower for women than for men; standard deviation was greater among women in both countries.

**Table 2:** Outcome and covariates distribution by gender and country.

Outcome:	*BMI*	*Women*		*Men*	
		USA	CANADA	USA	CANADA
	Mean	27.2	25.3	28.0	26.7
	SD	6.4	5.4	5.4	4.5
	Skewness	2.2	2.0	2.3	1.6
**Age %**					
	[18–30[	16.1%	19.4%	19.3%	21.3%
	[30–45[	29.3%	25.8%	31.1%	27.0%
	[45–65[	34.6%	36.7%	34.5%	36.8%
	[65+	20.0%	18.1%	15.1%	14.9%
**Income %**					
Missing		12.9%	16.4%	10.2%	13.0%
Lowest		22.4%	18.1%	25.9%	18.6%
Low		19.0%	16.3%	17.4%	23.2%
High		25.4%	28.1%	26.9%	22.9%
Highest		20.3%	21.1%	19.6%	22.3%
**Education %**					
No High School		9.6%	14.0%	10.4%	13.7%
HS Diploma		27.7%	17.4%	28.5%	16.5%
College		28.2%	44.5%	24.7%	45.7%
Graduate studies		34.5%	24.1%	36.4%	24.1%
**Race%**					
	White	70.7%	82.8%	69.6%	82.9%
	Asian	2.8%	11.2%	3.7%	10.7%
	Black	11.0%	2.4%	9.4%	2.3%
	Other	15.5%	3.6%	17.3%	4.1%
**Urbanity%**					
	Urban	88.1%	82.8%	88.2%	82.0%
	Other	8.3%	17.2%	8.2%	18.0%
	Unknown	3.6%	-	3.6%	-

### Independent variables

Independent variables were age, income, educational attainment, race and living in an urban environment. Table 2 presents the distribution of samples by all covariates. Household income was adjusted for household size by dividing the income by the square root of the household size (i.e. household size equivalized income) [32]. We then created quartiles of income by gender and sample year (2009 and 2010), and added the missing income category.

Since academic systems between and within countries are not uniform, educational attainment categories were based on the number of completed school years, and on diplomas earned. In the USA, the “No high school diploma” category includes those who never went to school up to those who stopped after 11 years of schooling; in Canada, this category includes also those who had been thirteen years at school but had no diploma. The “high school” category includes grade 12 or the equivalent in the USA; in Canada, this category includes only those who successfully finished high school (secondary-5 diploma or 13^th^ year completed). The “college” category includes those who had completed one to three years of college or technical school in the USA; in Canada it includes all those who did some post-secondary, with or without a college diploma, including those who received a university certificate (only one year at the university). Finally in the USA, the “graduate studies” category includes at least four years spent at college and graduation; in Canada, this category includes those with a baccalaureate diploma or higher. We used the same educational attainment category label, although slight differences in their meaning exist between both countries.

The CCHS did not provide the same level of detail for race/ethnicity characteristics than the BRFSS. Race was consequently categorized into only four groups: Whites, Blacks, Asians, and others.

In the USA, urban environment was assessed at the county-level by the US Department of Agriculture [33]. A county with more than 20,000 people was considered as urban. This information was missing for 3.6% of the sample. In Canada, an urban environment was assessed using the primary and secondary urban core, which are both characterized by a demographic concentration of 1000 individuals and contain 400 individuals/km^2^ (no missing information). Other areas were assumed to be non-urban.

### Analysis

Constraints affecting people's everyday opportunities to make health a priority may vary by gender [34]. Socially constructed body weight norms and ideas often differ between men and women within a given society, and so might social disparities in body weight [35]. All analyses were therefore stratified by gender in addition to being stratified by country, thus giving four subsamples for which findings could be compared. In line with the objectives of the study, we used a four-step procedure. Greater details in the structure of the multilevel models, the geographic variance estimation, the individual parameters estimation, and the estimated BMI computed out of a three level variance-covariance matrix are given in File S1.

#### 1 Hierarchical distribution of BMI variance

The first step aimed to estimate the distribution of BMI variance between the three hierarchical levels, and to estimate the proportion of variance explained by individual-level demographic and SES characteristics at each level. To do so, we first constructed an empty multilevel model without any covariates, known as the *null model*. This allowed partitioning the variance between the three levels. We then built a fully *adjusted model* which controlled for age, race, income, education and living in an urban environment. Reference categories were youngest age group, highest household income level, graduate studies, white race and urban setting. The variance structure was described using two indexes: the *variance partition coefficient* (VPC) and the *level-specific change in variance* (*Δσ^2^*). The VPC measures the proportion of variance for the geographic levels (subnational and regional combined) within one model; the level-specific change in variance measures the proportion of change in variance for each level between the null and the adjusted model. Taken together, these indexes describe how much of the variation is explained by the variables included in the model. Because of the nature of the geographical units, comparison of these indexes is more precise within a country than between them.

#### 2 Association of BMI with individual socioeconomic status

Using the fully adjusted model, we analyzed the mean BMI and its 95% confidence interval for all income and education categories while controlling for other independent variables.

#### 3 Residuals analysis of subnational units

We further used the subnational-level residuals (*i.e.*, states in the USA and provinces in Canada) and associated standard error to plot and rank the mean BMI and the 95% CI for each sub-national unit. This procedure allowed a visualization of which units are significantly different from the national mean.

#### 4 Geographic variation of BMI differentials within the socioeconomic status

We allowed the slope to vary for each of the three levels. This type of model is typically called random-intercept-random-slope model. However in this case, since covariates were categorical, we call it a random-intercept and differential-variance multilevel model. The objective of building such a model is to produce a precise estimate of BMI and its variation for a specific SES category at a specific level, while controlling for the variance of all other SES categories at each level. Moreover, this model controlled the global autocorrelation of the geographical units anywhere within the 3^rd^ level. Because the “slope” varied simultaneously at each level by SES categories, any remaining spatial effect between units would not be directly associated with SES. We used this model to plot the BMI coverage bounce (a range that includes 95% of the observations) at the subnational and regional levels, for each category of income and education by gender and country.

All analyses were performed using MlwiN 2.27 with the iterated generalized least squares (IGLS) estimation method and the standardized sampling weights provided by CDC or Statistics Canada.

## Results

### 1 Hierarchical distribution of BMI variance

Table 3 presents the distribution of the variance for the subnational, regional, and individual levels. Most of the variance was at the individual level. The variance partition coefficient (VPC) showed that the proportion of BMI variation at the geographic levels was slightly higher for women in the USA than in Canada (4.0% and 3.3% respectively). For men, the difference was greater in the USA (4.9%) than in Canada (2.4%). When adjusting for age, race, education, income and living in an urban environment, about 1% of this higher-level variance was explained among women in both countries. This represents the portion of the BMI variance distributed at the regional and subnational levels that is explained by the geographic distribution of age, race, education, income and living in an urban environment. For men, adding these variables explained only 0.4% of the contextual variance in the USA and 0.9% in Canada.

**Table 3:** Variance partition and specific-level change in variance in the USA and Canada.

	USA			Canada		
Variance components	Null	Adjusted		Null	Adjusted	
	Coeff.(SE)	Coeff.(SE)	Δσ2	Coeff.(SE)	Coeff.(SE)	Δσ2
***Women***						
Sub-national	0.40 (0.09)	0.17 (0.04)	−57.5%	0.53 (0.23)	0.38 (0.16)	−28.3%
Regional	1.27 (0.13)	0.93 (0.09)	−26.8%	0.42 (0.11)	0.16 (0.04)	−61.9%
Individual	39.95 (5.18)	37.11 (4.70)	−7.1%	27.92 (2.26)	26.20 (2.06)	−6.2%
Spatial levels VPC	4.0%	2.9%		3.3%	2.0%	
***Men***						
Sub-national	0.13 (0.04)	0.10 (0.04)	−23.1%	0.21 (0.10)	0.16 (0.07)	−23.8%
Regional	1.30 (0.26)	1.16 (0.23)	−10.8%	0.28 (0.08)	0.13 (0.03)	−53.8%
Individual	27.84 (3.74)	26.60 (3.57)	−4.5%	19.94 (1.50)	18.92 (1.43)	−5.1%
Spatial levels VPC	4.9%	4.5%		2.4%	1.5%	

However, change in VPC was not identical between subsamples. Although the level-specific changes (*Δσ^2^*) were relatively similar between countries at the individual level (4.5% to 7.1%), much more heterogeneity was found at the geographic levels. Reduction in variance at the subnational level was greater in the USA (57.5% for women and 23.1% for men) than in Canada (28.3% and 23.8% respectively). At the regional level, reduction in variance was greater in Canada (61.9% and 53.8%) than in the USA (26.8% and 10.8%).

### 2 Association of BMI with individual socioeconomic status

**Figure 1:**
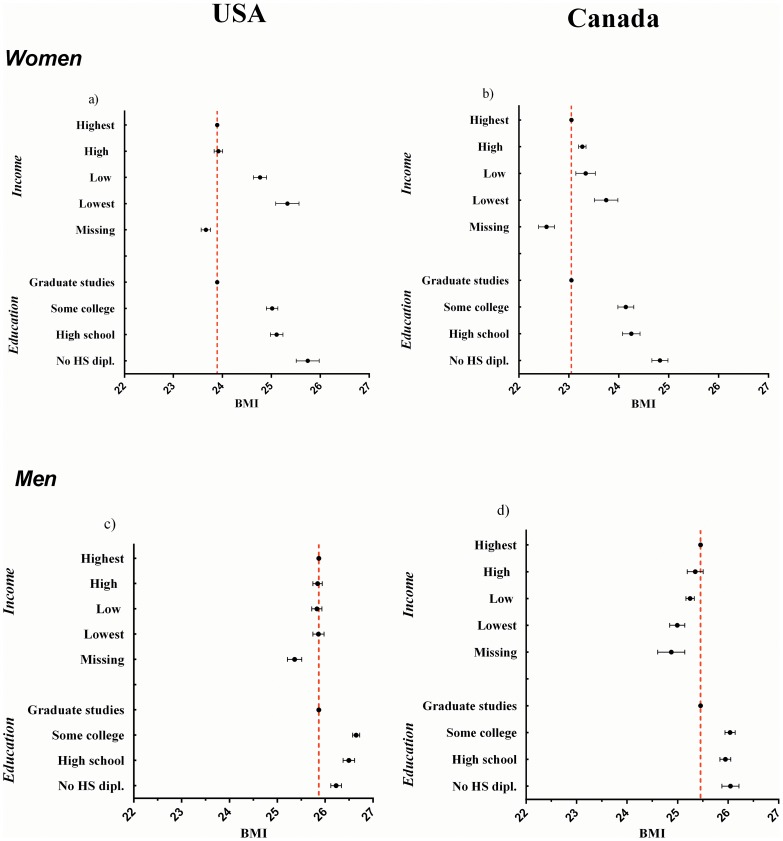
BMI by income and education for women and men in the USA and Canada, 2009.

Figure 1 shows the 95% CI of BMI means by education and income category. The reference category (young, highest income, graduate studies, White, living in an urban environment) had a higher BMI in the USA than in Canada, both for women and men. For women, associations were similar in both countries: the lower the household income or education level, the higher the BMI. This gradient, however, was stronger in the USA.

The situation was very different for men. The average BMI of the reference category was about two points higher than for women in both countries. Income was *not* associated with BMI in the USA, and a positive association was observed in Canada –higher income levels translating into higher BMIs. Concerning the education level, an inversed U-shaped relation was observed in the USA where respondents with graduated studies and those without high school diploma had the lowest BMI, while those with some college education had the highest (Figure 1c). In Canada, only the most educated men had a slightly, but significantly, different BMI than all other categories, with an average 0.5 kg/m^2^ lower.

### 3 Residuals analysis of subnational units

**Figure 2:**
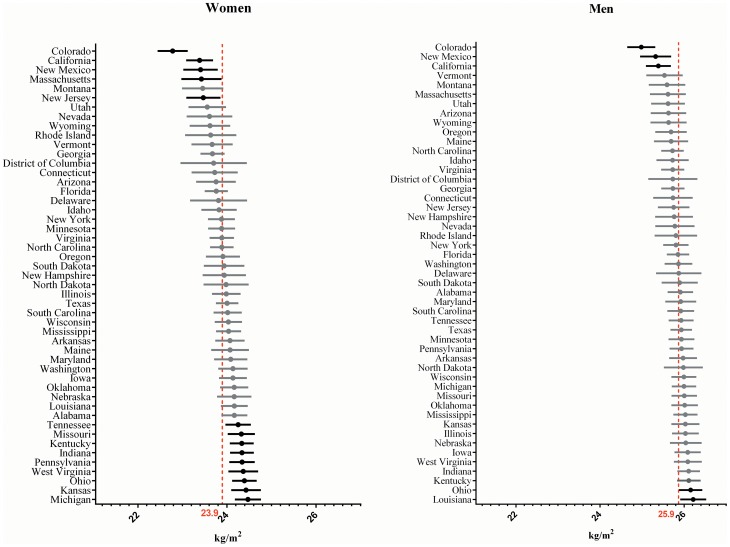
BMI mean and 95% confidence interval for US states, 2009 Reference group: young adult, White, highest household income and college graduated.

Regression residuals allowed the estimation of the sub-national level mean BMI. Figure 2 presents American states ranked by the reference category's BMI average. Based on the 95% CI, three states had a significantly lower BMI than the national average for men, - Colorado, New Mexico, and California-, and two states had a significantly higher BMI, -Ohio and Louisiana. These states appeared in similar positions in the caterpillar plot for women, except for Louisiana, which was not significantly different from the national average. An additional nine states showed a significant difference in BMI average for women (Figure 2).

**Figure 3:**
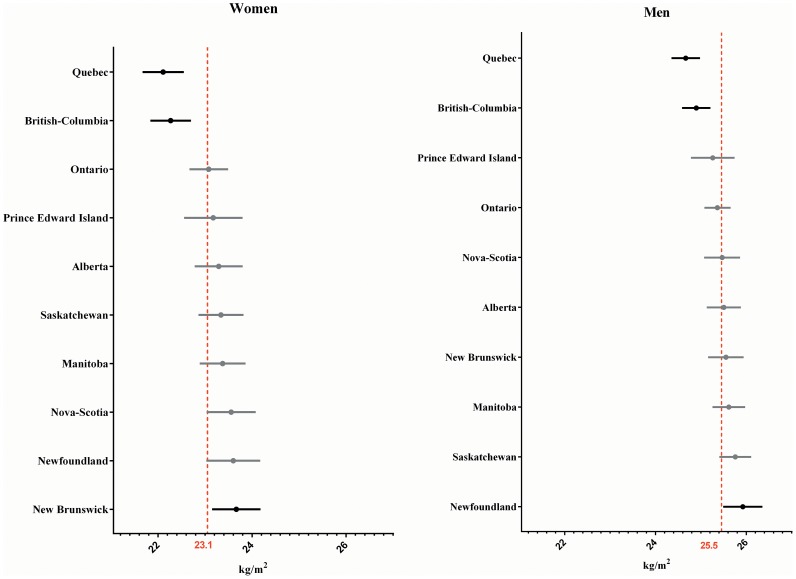
BMI mean and 95% confidence interval for Canadian provinces, 2009. Reference group: young adult, White, highest household income and college graduated.

Among the ten Canadian provinces (Figure 3), only Quebec and British Columbia showed a significantly different – and lower – BMI average for both women and men. Men in Newfoundland presented a significantly higher BMI than the national average, while for women this was true only in New-Brunswick.

### 4 Geographic variation of BMI differentials within the socioeconomic status

We used the complete model which included all covariates and where a variance-covariance matrix was produced for each level. This was performed separately for women and men, income and education, and for the USA and Canada (eight models).

**Figure 4:**
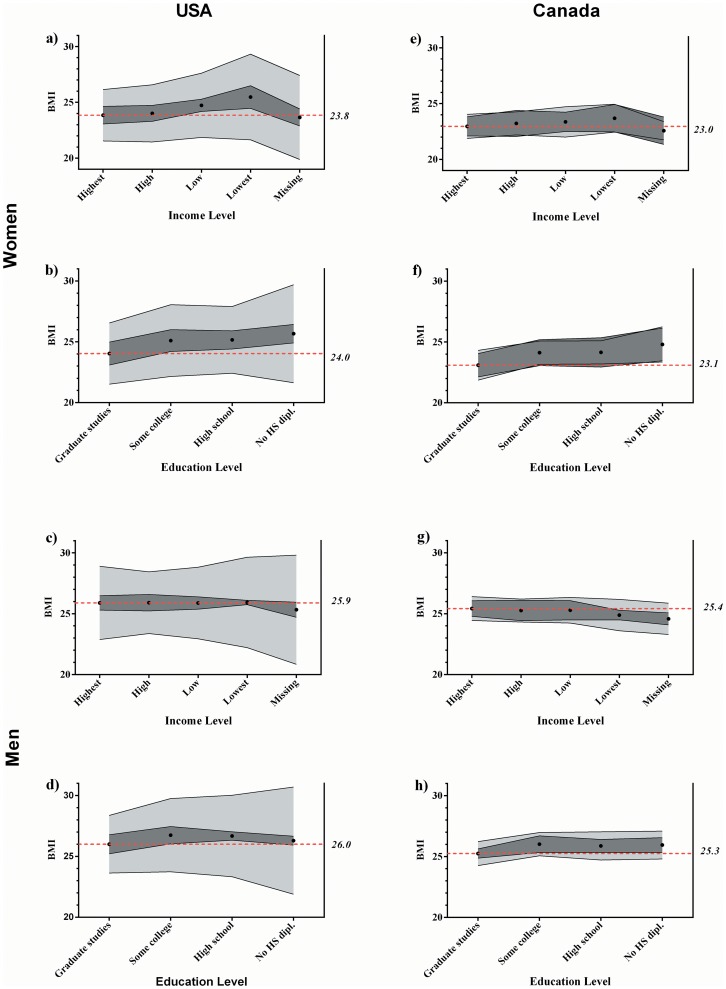
BMI range by income and education category for women and men in the USA and Canada, 2009. Dark grey = subnational-level; light grey = regional-level.

Figure 4 presents the average BMI and the 95% coverage bounds (CB) by education and income categories for subnational (dark grey areas) and regional levels (light grey areas). The CB shows the range into which 95% BMI averages for all groups of the same level is likely to be. This figure synthesizes a lot of information and allows the comparison of patterns in BMI variance for both geographic levels (subnational and regional) by SES, gender, and country. For example, range of the estimated mean BMI for American women in the highest income category (Figure 4a) ranged from 23.1 to 24.6 kg/m^2^ at the state level (range = 1.5 kg/m^2^). Following the dark gray area, we noticed a rise in the BMI mean but very little variation in the 95% CB between income categories. Indeed, the lowest income group ranged from 24.5 to 26.5 (range = 2 kg/m^2^) showing that although the mean BMI is rising for lower SES groups, variation around the mean remains constant. This means that when controlling for within states variation between counties, the average BMI of each state is fairly similar for all income categories. The picture is very different at the regional-level. Around the same BMI mean for the highest income group, we observed the CB going from 21.6 to 26.2 kg/m^2^ (range = 4.6 kg/m^2^) and from 21.7 to 29.3 kg/m^2^ (range = 7.6 kg/m^2^) for the lowest income groups. This means that in a given state, the average BMI of rich women in each county varies by 4.6, whereas average BMI of the poor ones vary by 7.6. There is much more variation at the county-level and the range becomes wider for the lower income groups. Similar observations were made for education level (Figure 4b). While the BMI means vary little by educational attainment levels between states, the variations between counties is much more important, and particularly for low education groups. Put differently, women with a higher education tend to have more similar BMIs, whatever the county they live in (more homogeneity), while those with a lower education have more different BMIs from one county to another (more heterogeneity).

We observed a similar variance pattern in BMI distributions for USA men; less variation between SES categories at the state-level, and an augmentation of the CB around the average for those with a lower disposable income or educational attainment (Figures 4c and 4d). The variation was more important than for women at the county-level, but the variation between states was much less noteworthy. This indicates that although the global variation between levels was significantly different, the BMI variation specifically for lower SES men was almost entirely explained by local variations, very little variance between states was remaining.

The observations were very different in Canada (Figures 4e to 4h). The geographic variance was found to be significant at all levels, but globally less important than in the USA. We also observed that the variance was relatively similar by SES (income and education) and between geographic levels. No clear pattern of variance by SES category was detected for BMI distribution of Canadians.

## Discussion

The overall objective of this study was to explore SES-BMI associations in the USA and in Canada for women and men, and the geographic variability of such associations by SES. Analyses of the distribution of the BMI variance at the subnational, regional, and individual levels revealed geographic differences in the BMI variance distributions between higher levels in both countries, with a variance partition coefficient (VPC) ranging from 2.4% and 4.9%. Adjusting for age, race, income, educational attainment and living in an urban environment, the level-specific change in variance (*Δσ^2^*) was more important at the regional and subnational levels (geographic levels) than at the individual level. This suggests that covariates like household income and educational attainment were unequally distributed between territories. Consideration of multiple aggregation levels (*i.e.* geographic autocorrelation within levels) revealed significant geographic variation in BMI within both countries. Concerning women in the USA for example (Table 3), the difference between the null and the adjusted models suggest that most of the variation observed at the sub-national level was explained (57.5%) as well as a considerable part at the regional level (26.8%). Between the two models, only 1.1% of the total variance was explained this way which leave 2.9% that remains to be explained by other influences which is primarily attributed to the regional level. For men, very little of the geographic variation is explained by individual characteristics (0.4%), and the remaining variance (4.5%) is also mainly attributed to the regional level. In Canada, the geographic variation of BMI between the null and the adjusted models was more important among women than men. The part of this variation explained by the individual characteristics mainly contributed to reduce the regional-level variation leaving respectively 2.0% and 1.5%.

Comparing the between-level variance within a country is very informative. It illustrates that BMI is not homogeneously distributed between sub-national units and regions, and at which geographic level this phenomenon tends to concentrate. However, comparing the variance between countries is not straightforward. The actual metrics used may not always be comparable due to the fact that they are directly issued from the geographic structure of administrative units specific to each country. As noted earlier, the hierarchical structure made by counties nested in states in the USA, may represent a different context than in the health region-province structure in Canada, regarding how they are occupied by the population and how interventions are implemented.

Nevertheless, since exactly the same statistical procedure was used, comparing the association of BMI with individual socioeconomic status was more straightforward. The observed negative association between SES and BMI for women had been reported before in developed countries [11], including USA and Canada [12], [13]. However, a clear gradient was only found between BMI and income in US women. Describing these associations as a gradient may be true only for women, although intermediate categories are not significantly different from each other. Associations in men were less straightforward and were different in the two countries: income was positively associated with BMI among Canadians and not significant in the USA; education had a non-linear association among Americans, and was not significant for Canadians.

Residual analysis of subnational units may provide additional information and may help to explain previous observations of variance distribution. The prevalence of obesity has been reported to be globally higher in the USA than in Canada for both genders [6], [25], [36]. This may not be true at the sub-national level however. Third-level residual plots (Figure 3 and 4) show where this variation is located within countries, and thus illustrate which areas tend to have higher and lower mean BMI for the reference category. For example, women in the state of Colorado, in the USA, were found to have an average BMI of 22.8, while women from New-Brunswick in Canada have an average BMI of 23.7. The sub-national units that significantly differ from the national average may host a very specific situation and therefore hide a part of the unexplained variation. These observations provide rationale for further investigation on the socioeconomic and geographic distribution of BMI.

The last step of our study aimed to further explore this “sociogeographic” distribution of BMI with the use of random intercepts and differential variance models. These models simultaneously disclosed the variation between the two geographic levels of aggregation as a function of SES, while controlling for SES heterogeneity at the individual level (differential of variance between groups). Releasing the variation in the BMI means by geographic context and SES revealed more than the averages alone could (Figure 4).

First, more BMI variance was observed at the regional than at the subnational level in the USA, while the difference between these levels was generally small in Canada. This suggests that more investigations should be done at the regional-level to understand how BMI is spatially distributed in the USA. Second, approximately the same variation is explained by individual characteristic between women and men in Canada (close to 1%). In the USA, however, almost no variation was explained by these characteristics in men's BMI while 4.5% of the variation remains unexplained. This suggests that socioeconomic characteristics among men may be less geographically clustered than women, and that BMI geographic distribution may be explained by other factors. Third, differences in mean BMI between regions were larger when socioeconomic status decreased at the regional-level in the USA. That is, in a given US state, and when holding age, gender, race and living in an urban environment constant, the mean BMI of low SES individuals has a higher variance than other SES categories. This SES gradient in the **variation** of BMI is particularly important between educational categories (Figure 4b and 4d). The gradient was not observed in Canada. Although the mean BMI varies by SES and gender, the variation of BMI by SES and gender is relatively constant at all levels.

These research findings reveal that studying the BMI distribution through the county-state hierarchy allows the detection of social disparities in the USA. Results also suggest a contextual effect [37] in the USA concerning lower SES individuals at the county-level and that these disparities may be addressed within the current administrative structure. In Canada, however, this is less obvious. There are significant geographic differences but less remained unexplained after we controlled for SES.

To our knowledge, this is the first USA-Canada comparative study that provides a deep insight into SES associations with BMI. This study shows that analysis of the averages alone may not be sufficient to understand how the phenomenon varies. Although contextual variances could be considered as relatively “modest,” they may have potentially important policy implications for population health [38], [39]. For example, why does BMI vary more between regions as a function of SES in the USA than in Canada? Is it really only a matter of the geographic distribution of administrative structures? Or is this revealing larger weight-related sociogeographic inequalities in the USA? Does the unequal variance between SES suggest that subgroups of the population do not necessarily respond homogeneously to the same environment and that some are particularly vulnerable to some contextual characteristics? Might the observed contextual effect translate into different body weights for men and women, and create different patterning in obesity through space? Results of this study suggest that this is more likely to be the case in the USA than in Canada and highlight the need to keep these questions in mind when defining weight related public heath interventions. Our results also suggest that a change in the mean does not necessarily reflect the change in the variance. Since a change in the variance may be a symptom of a social mechanism affecting health for a specific subgroup of the population, analyzing the variance by SES may be a good way to detect subtle influences of social forces underlying social inequalities.

Even though describing the obesity phenomena in terms of social gradients may be misleading at the individual level [3], [7], [10], [11], sociogeographic disparities follow a clear gradient at the regional level in the USA. This gradient in regional variance is not present in Canada however. Yet it is possible these patterns may be linked to local differences in the spatial distribution of resources promoting active living and healthy eating or other resources associated with the adults' weight status [21]. Other hypotheses could point towards other more global differences in national culture, urban development, economic regulation, or social policies, and warrant further investigation.

Statistical analyses using geographical units is always subject to what is called the modifiable areal unit problem (MAUP), which is the uncertainty about which geographical set of units to use for analysis [41]. This refers more specifically at the effect of changing the scale of observation or the aggregation criterion. To improve the modelling strategy, the use of smaller geographic units may provide a more detailed description of the phenomena in Canada and may reveal hidden disparities. It is possible that the scale of analysis we used (the health regions) is inappropriate; however, many public heath interventions are applied at this level. On the other hand, in the USA, the important variations detected between and within SES groups at the state and the county scale suggest this hierarchy may be appropriate for the analysis of the geographic distribution of a public health phenomenon like obesity. Beyond the MAUP, further investigations encompassing not only SES and geographic variation, but also time variation are essential to answer such questions [40].

Several limitations need to be kept in mind when interpreting results. International comparative studies are always challenging and need more flexibility than other studies. Details in measurements may rarely be exactly the same. However, during variable construction it was important to make measures as comparable as possible. BRFSS and CCHS are cross-sectional surveys using self-reported information and therefore cannot be used to infer causality. Associations between income and BMI for US men may be inaccurate since some interaction was found between the “high income” category and the “missing income”. This interaction, however, did not change other associations or the distribution of variance between levels. Although this investigation mainly focused on income, education and geography, it considered only a limited number of explanatory factors. Other measurements concerning the geographic contexts such as foodscapes, urban form, or neighborhood-level SES [21] might also be very informative. Because of the small number of provinces included in Canada (n = 10), the variance at this level might be too constrained, which may limit our ability to compare findings at the subnational level. Identifying the underlying causes that would explain the geographic variations between the USA and Canada are beyond the scope of this paper. The exploratory and comparative nature of the study provided good support for further investigation on this matter. Nevertheless, despite these limitations, we are confident that our results could help to point out helpful differences in the BMI distribution patterns between countries.

The rise in obesity may be described as a natural biological response of individuals to a changing society [42]. However, this response may greatly vary and depend of the distribution of a diversity of sociogeographic contexts. The scientific literature provides evidence of socioeconomic patterning of obesity, and recommends that prevention initiatives better take into account SES and geographic stratifications [11], [43]. The results of our research are in line with this suggestion.

Our exploratory analyses provided new insights on the geographic variability of the association between BMI, education, and income in the USA and in Canada, as well as the distribution of its associated variance. This paper shows how SES-BMI associations vary geographically beyond individual level associations. The current knowledge on obesity would particularly benefit from a repeated cross-sectional or longitudinal application of this modeling strategy. This could reveal how the variance in SES-BMI associations evolves through both space and time.

## Supporting Information

File S1 Analyses using multilevel models with three level variance-covariance matrixes.
(DOC)
