# Supplementary material for: Geographic Variability in the Association between Socioeconomic Status and BMI in the USA and Canada
Source: PLoS One. 2014 Jun 16;9(6):e99158. doi: 10.1371/journal.pone.0099158 (PMC4059636; doi:10.1371/journal.pone.0099158)
Supplement: File S1 — Analyses using multilevel models with three level variance-covariance matrixes. (DOC) [file pone.0099158.s001.doc]

Supporting Information

**S1. Analyses using multilevel models with three level variance-covariance matrixes**

In line with the objectives of the study, we used a four-step procedure.

The first step aimed to estimate the distribution of the BMI variance between the three hierarchical levels, and to estimate the proportion of variance explained by individual-level demographic and SES characteristics at each level. To do so, we first constructed an empty three-level model - without any covariate, known as the *null model* [1] - included three error terms in the random part (one per level). A fully *adjusted* *model* [2] controlled for age, race and urban density (
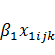
), while the focus was on income and education (
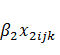
). Reference categories were youngest age group, highest household income level, graduate studies, white race and high urban density.

[1]

[2]

The variance structure was described using two indexes: the *variance partition coefficient* (VPC) [3] and the *level-specific change in variance* (*Δσ^2^*) [4]. The VPC measures the proportion of variance for the spatial levels (subnational and regional combined) within one model; the level-specific change in variance measures the proportion of change in variance for each level between the null and the adjusted model.

[3]

[4]

The second step analyzed the mean BMI and its 95% confidence interval (CI) for each category of income and education, while controlling for age and race (fully adjusted model [2]).

For the third step, we used subnational-level residuals (*i.e.,* states in the USA and provinces in Canada) and their standard error to obtain mean BMI and 95% coverage bounds for each region.

In the last step, all levels could vary as a function of income or education (in eight distinct models with error terms
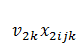
,
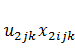
and
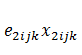
in the random part [5]). This type of model is typically called random-intercept-random-slope model, however in this case, since covariates were categorical we called it random-intercept and differential-variance multilevel model. Ranges of BMI at the subnational and regional levels were plotted by category of income and education by gender and countries.


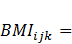


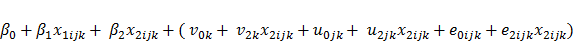
 [5]

All analyses were performed using MlwiN 2.27 with the iterated generalized least squares (IGLS) estimation method and the standardized sampling weights provided by CDC or Statistics Canada.

Table S1. Null model for women, USA 2009-2010

| **Fixed Part** | **Constant (S.E.)** |
| --- | --- |
|  | 27.368 (0,098) |
| **Random Part** | **Var (S.E.)** |
| Level: subnational | 0.395 (0,086) |
| Level: region | 1.272 (0,127) |
| Level: individual | 39.953 (5,184) |
| VPC (Spatial levels ) | 4.0% |
| -2*loglikelihood: | 3537085.088 |

Table S2. Null model for men, USA 2009-2010

| **Fixed Part** | **Constant(S.E.)** |
| --- | --- |
|  | 28.215 (0,062) |
| **Random Part** | **Var (S.E.)** |
| Level: subnational | 0.128 (0,041) |
| Level: region | 1.303 (0,259) |
| Level: individual | 27.831 (3,744) |
| VPC (Spatial levels ) | 4.9% |
| -2*loglikelihood: | 2179357.036 |

Table S3. Null model for women, Canada 2009-2010

| **Fixed Part** | **Constant(S.E.)** |
| --- | --- |
|  | 26,155 (0,248) |
| **Random Part** | **Var (S.E.)** |
| Level: subnational | 0,528 (0,226) |
| Level: region | 0,422 (0,11) |
| Level: individual | 27,922 (2,262) |
| VPC (Spatial levels ) |  |
| -2*loglikelihood: | 3.3% |

Table S4. Null model for men, Canada 2009-2010

| **Fixed Part** | **Constant(S.E.)** |
| --- | --- |
|  | 27,244(0,165) |
| **Random Part** | **Var (S.E.)** |
| Level: subnational | 0,211 (0,097) |
| Level: region | 0,282 (0,075) |
| Level: individual | 19,939 (1,496) |
| VPC (Spatial levels ) |  |
| -2*loglikelihood: | 2.4% |

**Table S5. Adjusted model for women, USA 2009-2010**

| **Fixed Part** | **Beta (S.E.)** |
| --- | --- |
| cons | 23,895 (0,115) |
| young | 1,913 (0,082) |
| old | 2,718 (0,091) |
| oldest | 1,387 (0,093) |
| asian | -2,094 (0,156) |
| black | 2,992 (0,1) |
| other | 1 (0,138) |
| cycle_10 | 0,07 (0,031) |
| missing_income | -0,232 (0,048) |
| lowest_income | 1,433 (0,122) |
| low_income | 0,875 (0,067) |
| high_income | 0,024 (0,044) |
| No_HS_diploma | 1,849 (0,12) |
| High school | 1,214 (0,064) |
| some college | 1,123 (0,06) |
| region_unknown | -0,373 (0,119) |
| rural | 0,148 (0,049) |
| **Random Part** | **Var (S.E.)** |
| Level: subnational | 0,171 (0,044) |
| Level: region | 0,925 (0,093) |
| Level: individual | 37,11 (4,699) |
|  |  |
| VPC (Spatial levels ) | 2,9% |
|  |  |
| -2*loglikelihood: | 3501129,693 |

**Table S6. Adjusted model for men, USA 2009-2010**

| **Fixed Part** | **Beta (S.E.)** |
| --- | --- |
| cons | 25,868 (0,1) |
| young | 2,285 (0,069) |
| old | 2,546 (0,074) |
| oldest | 1,366 (0,067) |
| asian | -2,167 (0,155) |
| black | 0,514 (0,08) |
| other | 0,669 (0,068) |
| cycle_10 | 0,058 (0,044) |
| missing_income | -0,506 (0,076) |
| lowest_income | -0,007 (0,059) |
| low_income | -0,04 (0,055) |
| high_income | -0,025 (0,049) |
| No_HS_diploma | 0,362 (0,057) |
| High school | 0,627 (0,061) |
| some college | 0,78 (0,038) |
| region_unknown | -0,059 (0,128) |
| rural | 0,234 (0,057) |
| **Random Part** | **Var (S.E.)** |
| Level: subnational | 0,096 (0,037) |
| Level: region | 1,159 (0,233) |
| Level: individual | 26,606 (3,568) |
|  |  |
| VPC (Spatial levels ) | 4,5% |
|  |  |
| -2*loglikelihood: | 2165297,326 |

**Table S7. Adjusted model for women, Canada 2009-2010**

| **Fixed Part** | **Beta (S.E.)** |
| --- | --- |
| cons | 23,052 (0,225) |
| young | 1,811 (0,08) |
| old | 2,588 (0,115) |
| oldest | 2,069 (0,14) |
| asian | -1,652 (0,109) |
| black | 0,857 (0,06) |
| other | 0,174 (0,184) |
| cycle_10 | 0,066 (0,044) |
| missing_income | -0,5 (0,081) |
| lowest_income | 0,695 (0,12) |
| low_income | 0,285 (0,1) |
| high_income | 0,219 (0,038) |
| No_HS_diploma | 1,772 (0,082) |
| High school | 1,199 (0,09) |
| some college | 1,09 (0,081) |
| Not urban | 0,176 (0,085) |
| **Random Part** | **Var (S.E.)** |
| Level: subnational | 0,382 (0,164) |
| Level: region | 0,162 (0,043) |
| Level: individual | 26,197 (2,06) |
|  |  |
| VPC (Spatial levels ) | 2,0% |
|  |  |
| -2*loglikelihood: | 355539,483 |

**Table S8. Adjusted model for men, Canada 2009-2010**

| **Fixed Part** | **Beta (S.E.)** |
| --- | --- |
| cons | 25,454 (0,129) |
| young | 1,62 (0,118) |
| old | 2,098 (0,044) |
| oldest | 1,357 (0,116) |
| asian | -1,756 (0,079) |
| black | -0,182 (0,368) |
| other | 0,033 (0,281) |
| cycle_10 | 0,155 (0,045) |
| missing_income | -0,58 (0,137) |
| lowest_income | -0,462 (0,077) |
| low_income | -0,207 (0,042) |
| high_income | -0,106 (0,08) |
| No_HS_diploma | 0,592 (0,086) |
| High school | 0,491 (0,054) |
| some college | 0,584 (0,051) |
| Not urban | 0,3 (0,097) |
| **Random Part** | **Var (S.E.)** |
| Level: subnational | 0,156 (0,074) |
| Level: region | 0,125 (0,034) |
| Level: individual | 18,917 (1,43) |
|  |  |
| VPC (Spatial levels ) | 1.5% |
|  |  |
| -2*loglikelihood: | 283143,785 |

**Table S9. Mean BMI and coverage bounds for wo*men* per *income*** category and by level, USA 2009-2010

| **Level** | **mean** | **CB 95%** | | |
| --- | --- | --- | --- | --- |
|  |  | **Lower** | **Higher** | |
| **Subnational**  Highest | 23,85 | 23,06 | 24,63 |  |
| High | 24,01 | 23,30 | 24,73 |  |
| Low | 24,73 | 24,18 | 25,28 |  |
| Lowest | 25,48 | 24,46 | 26,49 |  |
| Missing | 23,65 | 22,89 | 24,40 |  |
|  |  |  |  |  |
| **Region** |  |  |  |  |
| Highest | 23,85 | 21,54 | 26,15 |  |
| High | 24,01 | 21,45 | 26,58 |  |
| Low | 24,73 | 21,85 | 27,61 |  |
| Lowest | 25,48 | 21,64 | 29,31 |  |
| Missing | 23,65 | 19,88 | 27,41 |  |
|  |  |  |  |  |
| **Individual** |  |  |  |  |
| Highest | 23,85 | 13,62 | 34,07 |  |
| High | 24,01 | 12,75 | 35,27 |  |
| Low | 24,73 | 13,14 | 36,32 |  |
| Lowest | 25,48 | 10,90 | 40,05 |  |
| Missing | 23,65 | 12,80 | 34,49 |  |

**Table S10. Mean BMI and coverage bounds for *men* per *income*** category and by level, USA 2009-2010

| **Level** | **mean** | **CB 95%** | | |
| --- | --- | --- | --- | --- |
|  |  | **Lower** | **Higher** | |
| **Subnational**  Highest | 25,88 | 25,29 | 26,47 |  |
| High | 25,90 | 25,23 | 26,57 |  |
| Low | 25,88 | 25,37 | 26,38 |  |
| Lowest | 25,92 | 25,74 | 26,10 |  |
| Missing | 25,32 | 24,70 | 25,94 |  |
|  |  |  |  |  |
| **Region** |  |  |  |  |
| Highest | 25,88 | 22,88 | 28,89 |  |
| High | 25,90 | 23,36 | 28,44 |  |
| Low | 25,88 | 22,93 | 28,82 |  |
| Lowest | 25,92 | 22,21 | 29,63 |  |
| Missing | 25,32 | 20,84 | 29,80 |  |
|  |  |  |  |  |
| **Individual** |  |  |  |  |
| Highest | 25,88 | 17,71 | 34,06 |  |
| High | 25,90 | 16,05 | 35,75 |  |
| Low | 25,92 | 17,16 | 34,68 |  |
| Lowest | 25,92 | 13,76 | 38,08 |  |
| Missing | 25,32 | 15,06 | 35,59 |  |

**Table S11. Mean BMI and coverage bounds for wo*men* per *income* category and by level, Canada 2009-2010**

| **Level** | **mean** | **CB 95%** | | |
| --- | --- | --- | --- | --- |
|  |  | **Lower** | **Higher** | |
| **Subnational**  Highest | 22,96 | 22,10 | 23,82 |  |
| High | 23,22 | 22,06 | 24,39 |  |
| Low | 23,36 | 22,49 | 24,23 |  |
| Lowest | 23,69 | 22,46 | 24,92 |  |
| Missing | 22,57 | 21,34 | 23,80 |  |
|  |  |  |  |  |
| **Region** |  |  |  |  |
| Highest | 22,96 | 21,88 | 24,04 |  |
| High | 23,22 | 22,19 | 24,26 |  |
| Low | 23,36 | 22,00 | 24,72 |  |
| Lowest | 23,69 | 22,44 | 24,94 |  |
| Missing | 22,57 | 21,75 | 23,39 |  |
|  |  |  |  |  |
| **Individual** |  |  |  |  |
| Highest | 22,96 | 13,77 | 32,15 |  |
| High | 23,22 | 12,21 | 34,23 |  |
| Low | 23,36 | 13,69 | 33,04 |  |
| Lowest | 23,69 | 13,43 | 33,95 |  |
| Missing | 22,57 | 13,08 | 32,06 |  |

**Table S12. Mean BMI and coverage bounds for *men* per *income* category and by level, Canada 2009-2010**

| **Level** | **mean** | **CB 95%** | | |
| --- | --- | --- | --- | --- |
|  |  | **Lower** | **Higher** | |
| **Subnational**  Highest | 25,42 | 24,78 | 26,06 |  |
| High | 25,26 | 24,42 | 26,10 |  |
| Low | 25,28 | 24,49 | 26,07 |  |
| Lowest | 24,88 | 24,49 | 25,27 |  |
| Missing | 24,57 | 24,08 | 25,07 |  |
|  |  |  |  |  |
| **Region** |  |  |  |  |
| Highest | 25,42 | 24,44 | 26,40 |  |
| High | 25,26 | 24,32 | 26,20 |  |
| Low | 25,28 | 24,23 | 26,33 |  |
| Lowest | 24,88 | 23,59 | 26,17 |  |
| Missing | 24,57 | 23,29 | 25,86 |  |
|  |  |  |  |  |
| **Individual** |  |  |  |  |
| Highest | 25,42 | 17,61 | 33,23 |  |
| High | 25,26 | 16,44 | 34,08 |  |
| Low | 25,28 | 16,72 | 33,84 |  |
| Lowest | 24,88 | 16,22 | 33,55 |  |
| Missing | 24,57 | 15,81 | 33,34 |  |

**Table S13. Mean BMI and coverage bounds for wo*men* per *education* category and by level, USA** 2009-2010

| **Level** | **mean** | **CB 95%** | |
| --- | --- | --- | --- |
|  |  | Lower | Higher |
| **Subnational** |  |  |  |
| Graduate studies | 24,03 | 23,09 | 24,97 |
| Some college | 25,10 | 24,21 | 25,99 |
| High school | 25,16 | 24,41 | 25,90 |
| No HS diploma | 25,66 | 24,90 | 26,42 |
|  |  |  |  |
| **Region** |  |  |  |
| Graduate studies | 24,03 | 21,52 | 26,54 |
| Some college | 25,10 | 22,15 | 28,05 |
| High school | 25,16 | 22,41 | 27,90 |
| No HS diploma | 25,66 | 21,63 | 29,69 |
|  |  |  |  |
| **Individual** |  |  |  |
| Graduate studies | 24,03 | 12,97 | 35,09 |
| Some college | 25,10 | 12,92 | 37,28 |
| High school | 25,16 | 13,41 | 36,90 |
| No HS diploma | 25,66 | 12,39 | 38,93 |

**Table S14. Mean BMI and coverage bounds for *men* per *education*** category and by level, USA 2009-2010

| **Level** | **mean** | **CB 95%** | |
| --- | --- | --- | --- |
|  |  | Lower | Higher |
| **Subnational** |  |  |  |
| Graduate studies | 25,99 | 25,21 | 26,77 |
| Some college | 26,74 | 26,03 | 27,45 |
| High school | 26,67 | 26,32 | 27,01 |
| No HS diploma. | 26,29 | 25,93 | 26,65 |
|  |  |  |  |
| **Region** |  |  |  |
| Graduate studies | 25,99 | 23,63 | 28,36 |
| Some college | 26,74 | 23,73 | 29,75 |
| High school | 26,67 | 23,32 | 30,01 |
| No HS diploma | 26,29 | 21,89 | 30,69 |
|  |  |  |  |
| **Individual** |  |  |  |
| Graduate studies | 25,99 | 17,27 | 34,71 |
| Some college | 26,74 | 16,38 | 37,09 |
| High school | 26,67 | 16,11 | 37,23 |
| No HS diploma | 26,29 | 15,10 | 37,49 |

**Table S15. Mean BMI and coverage bounds for wo*men* per education category and by level, Canada 2009-2010**

| **Level** | **mean** | **CB 95%** | |
| --- | --- | --- | --- |
|  |  | **Lower** | **Higher** |
| **Subnational** |  |  |  |
| Graduate studies | 23,08 | 22,13 | 24,04 |
| Some college | 24,12 | 23,06 | 25,18 |
| High school | 24,14 | 22,93 | 25,35 |
| No HS diploma | 24,80 | 23,45 | 26,14 |
|  |  |  |  |
| **Region** |  |  |  |
| Graduate studies | 23,08 | 21,86 | 24,30 |
| Some college | 24,12 | 23,16 | 25,07 |
| High school | 24,14 | 23,19 | 25,09 |
| No HS diploma | 24,80 | 23,35 | 26,24 |
|  |  |  |  |
| **Individual** |  |  |  |
| Graduate studies | 23,08 | 13,54 | 32,62 |
| Some college | 24,12 | 13,69 | 34,54 |
| High school | 24,14 | 14,02 | 34,26 |
| No HS diploma | 24,80 | 15,45 | 34,14 |

**Table S16. Mean BMI and coverage bounds for *men* per education category and by level, Canada 2009-2010**

| **Level** | **mean** | **CB 95%** | |
| --- | --- | --- | --- |
|  |  | **Lower** | **Higher** |
| **Subnational** |  |  |  |
| Graduate studies | 25,24 | 24,86 | 25,62 |
| Some college | 26,01 | 25,32 | 26,69 |
| High school | 25,87 | 25,34 | 26,40 |
| No HS diploma | 25,94 | 25,34 | 26,55 |
|  |  |  |  |
| **Region** |  |  |  |
| Graduate studies | 25,24 | 24,26 | 26,22 |
| Some college | 26,01 | 25,05 | 26,96 |
| High school | 25,87 | 24,71 | 27,02 |
| No HS diploma | 25,94 | 24,80 | 27,08 |
|  |  |  |  |
| **Individual** |  |  |  |
| Graduate studies | 25,24 | 16,48 | 34,00 |
| Some college | 26,01 | 17,49 | 34,52 |
| High school | 25,87 | 17,25 | 34,49 |
| No HS diploma | 25,94 | 17,91 | 33,98 |

**Table S17. Proportional difference in level variation between the null and adjusted models**

| **Null** | **Adjusted** |  | **Δσ^2^** |
| --- | --- | --- | --- |
| **USA women** | | | |
| 0.4 | 0.17 | -57.5 | -58% |
| 1.27 | 0.93 | -26.7717 | -27% |
| 39.95 | 37.11 | -7.10889 | -7% |
| **USA men** | | | |
| 0.13 | 0.1 | -23.0769 | -23% |
| 1.3 | 1.16 | -10.7692 | -11% |
| 27.84 | 26.6 | -4.45402 | -4% |
| **Canada women** | | | |
| 0.53 | 0.4 | -24.5283 | -25% |
| 0.42 | 0.18 | -57.1429 | -57% |
| 27.92 | 26.2 | -6.16046 | -6% |
| **Canada men** | | | |
| 0.21 | 0.17 | -19.0476 | -19% |
| 0.28 | 0.14 | -50 | -50% |
| 19.94 | 18.92 | -5.11535 | -5% |

Δσ^2^=((null- adjusted)/null)*100
